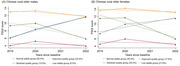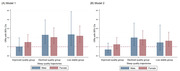# Sleep quality trajectories and motoric cognitive risk syndrome among Chinese rural older adults: Does gender matter?

**DOI:** 10.1002/alz.092723

**Published:** 2025-01-03

**Authors:** Dan Zhao, Bei Wu, Xiang Qi, Yaolin Pei, Tingting Gao, Jie Li, Yi Wang, Lina Wang, Chengchao Zhou

**Affiliations:** ^1^ Shandong University, Jinan, Shandong Province China; ^2^ New York University, New York, NY USA; ^3^ NYU Aging Incubator, New York, NY USA; ^4^ Yale University, New Heaven, CT USA; ^5^ Huzhou University, Huzhou, Zhejiang China; ^6^ NHC Key Lab of Health Economics and Policy Research (Shandong University), Jinan, Shandong Province China; ^7^ Institute of Health and Elderly Care (Shandong University), Jinan, Shandong Province China

## Abstract

**Background:**

Motoric cognitive risk syndrome (MCR), a pre‐dementia syndrome, is a risk factor for disability and mortality. However, few studies have examined the associations between sleep quality trajectories and MCR. This study aimed to explore the associations between sleep quality trajectories and MCR, and whether these associations vary by gender among Chinese rural older adults.

**Methods:**

We used three waves of follow‐up data (2019, 2020, and 2022) from the Shandong Rural Elderly Health Cohort (SREHC), including 2,168 participants aged 60 years or above. MCR was defined by the presence of subjective cognitive complaints and slow gait. The Pittsburgh Sleep Quality Index was used to assess sleep quality over the past month. We applied the group‐based trajectory modeling to identify latent groups and estimate sleep quality trajectories. Logistic regression models were performed to examine relationships between sleep quality trajectories and MCR by gender.

**Results:**

The prevalence of MCR was 13.9% among Chinese rural older adults. Four sleep quality trajectories among males and females were identified, including the normal stable group (male:55.4%; female: 35.4%), the improved quality group (male:23.4%; female: 17.6%), the declined quality group (male:13.0%; female: 25.2%), and the low stable group (male:8.3%; female: 21.8%). After adjusting for covariates, rural older females in the declined quality group (Odds Ratio [OR] = 1.81, 95% Confidence Interval [CI] = 1.19 ‐ 2.77) and the low stable group (OR = 1.65, 95% CI = 1.04 ‐ 2.60) were more likely to have MCR than those in the normal stable group. However, only the declined quality group (OR = 2.00, 95% CI = 1.10 ‐ 3.61) was associated with MCR among male rural older adults.

**Conclusion:**

Our findings suggest that declined sleep quality was associated with MCR among Chinese rural older males and females. Interventions are needed to focus on addressing sleep quality among rural older adults, which may help prevent MCR.